# Moffett's Solution Causes Significantly Greater Postoperative Throat Pain Compared to Cophenylcaine in Sinonasal Surgery

**DOI:** 10.1155/2019/3738647

**Published:** 2019-07-01

**Authors:** Dominic Ku, Kartik Vasan, Eugene Wong, Evan Tseros, Narinder Pal Singh

**Affiliations:** ^1^Department of Otolaryngology, Westmead Hospital, Sydney, New South Wales, Australia; ^2^University of Sydney, New South Wales, Australia

## Abstract

**Aim:**

Preoperative decongestion with Moffett's solution is routine practice in sinonasal procedures providing an ideal operative field. Anecdotally, it is related to postoperative throat pain, yet a quantitative relationship has not been established. We compare the incidence and severity of postoperative throat pain after application of Moffett's solution against Cophenylcaine decongestion.

**Methodology:**

A total of thirty patients from two consultants were recruited. The intervention arm (twenty) was decongested with Moffett's solution and the control arm (ten) with Cophenylcaine. The primary outcome was self-reported postoperative throat pain as measured by visual analogue scale (VAS) at 2 hours, 4 hours, 6 hours, and next morning.

**Results:**

There was a significantly higher VAS for throat pain in patients decongested with Moffett's solution in the early postoperative period (2 hours p=0.03, 4 hours p=0.04).

**Conclusion:**

Moffett's solution is associated with a greater severity of transient postoperative throat pain compared to topical Cophenylcaine. We recommend further studies to identify means to minimise this side effect.

**Clinical Trial Registration:**

This paper has been registered with the Australian and New Zealand Clinical Trials Registry under the registration number: ACTRN12619000772145.

## 1. Introduction

Moffett's solution is commonly used as a topical decongestant and local anaesthetic in sinonasal surgery. It is a combination of cocaine, adrenaline, bicarbonate, and 0.9% sodium chloride, which was first described by Major A. J. Moffett of the Royal Army Medical Corps in 1941 [1]. When applied topically to the nasal mucosa, it produces profound vasoconstriction and anaesthesia, reducing blood loss and improving visualisation in the operative field for sinonasal surgery [2, 3]. This combination of constituents has been shown to have greater efficacy than any of the individual components alone, and it has been noted to have an excellent safety profile when administered topically to the nasal mucosa in the prescribed manner [3–5].

In his 1941 paper “Postural Instillation”, Moffett described his method of instilling the solution via a syringe and a “Clarke's needle”, in which the patient's head is rotated through various positions to coat all of the mucosal surfaces [1]. In his second publication in 1947, Moffett had modified his solution to include 2mL of 8% cocaine (160mg), 2mL of 1% sodium bicarbonate, and 1mL of 1:1000 adrenaline [6]. In 2003, a UK survey reported that the majority (77%) of the 360 responding otolaryngologists used some form of topical cocaine, with the most common form being a mixture of 10% cocaine and 1:1000 adrenaline (48%) [7]. A US survey published in 2004 reported a trend of decreased cocaine usage in sinonasal surgery when compared to a 1977 survey, with the most often utilised solution including 4% cocaine on nasal pledgets [5, 8]. Although a range of cocaine concentrations are described, the agreed dosage for safe administration in the literature is 1.5mg/kg or a maximum of 200mg [2, 8]. Adverse events from cocaine use in nasal surgery are exceedingly rare—a large series of more than 100,000 patients by Feehan et al. reported a morbidity rate of 0.3% and mortality of 0.005% [9]. Cocaine is the popular choice of nasal preparation among rhinologists due to its ability to provide a superior operative field when compared to other agents [7].

Anecdotally, rhinologists from our unit and others have noted that significant postoperative throat pain is a common side effect of Moffett's solution use. However, this is underreported in the literature. To the best of our knowledge, there are no studies in the literature which assess the relationship between Moffett's solution and throat pain except one UK survey of otolaryngologists in 2010 which reported only four individual cases of throat pain from 159 otolaryngologists [4]. We hypothesise that the pain may arise, following Moffett's solution administration, from the pooling of the solution in the oropharynx, which may cause prolonged and profound vasoconstriction in the mucosa, resulting in localised ischaemia and subsequent throat pain. Our study aims to investigate and quantify this anecdotally observed effect to determine its presence, severity, and duration.

## 2. Methods

### 2.1. Setting

This multicentre prospective observational study was conducted at a Tertiary University Hospital in Sydney, Australia (Westmead Hospital, University of Sydney), in 2018.

### 2.2. Ethical Considerations

The study was approved by the Western Sydney Local Health District Human Research Ethics Committee (WSLHD HREC) as a low and negligible risk project (reference number: LNR/17/WMEAD/568). The WSLHD HREC is accredited by the New South Wales Ministry of Health to provide ethical and scientific review to conduct research within the NSW public health system. The study operates in accordance with the National Health and Medical Research Council's National Statement on Ethical Conduct in Human Research and the CPMP/ICH Note for guidance on Good Clinical Practice and the international guidelines for observational studies [10].

### 2.3. Participants

A consecutive series of patients undergoing routine sinonasal surgery (septoplasty, turbinate surgery, and functional endoscopic sinus surgery) requiring nasal preparation were included in the study. Patients with a history of previous sinonasal surgery were excluded from the study. Patients were recruited following informed consent.

### 2.4. Intervention

The treatment arms in this study were based on current practice by the two senior surgeons. Patients in the intervention arm (Moffett's solution) received topically applied Moffett's solution preoperatively via the MADgic™ atomiser (Teleflex Medical; Wayne, Pennsylvania, USA). The solution consisted of a mixture of 2mL of 10% cocaine solution (200mg), 1mL of 1:1000 adrenaline, 2mL of sodium bicarbonate, and 5mL of 0.9% sodium chloride solution, 10mL in total with 5mL applied to each side. Patients in the control arm received Cophenylcaine Forte™ topical nasal spray (ENT Technologies Pty Ltd.; Hawthorn East, Victoria, Australia), consisting of 5% lignocaine hydrochloride (50mg/ml, total 25mg) and 0.5% phenylephrine hydrochloride (5mg/ml, total 2.5mg) delivered using the provided atomised spray nozzle, five sprays per side (100 microlitre per spray). In both arms, the solution was applied following the induction of general anaesthesia, prior to surgery. The patients were blinded to the agent used, whereas the investigators applying the solution and recording the results were not.

### 2.5. Outcomes

The primary outcome for this study was throat pain measured by the patient recording a point on a 100mm visual analogue scale (VAS) for pain between 0mm (no pain) and 100mm (worst pain possible). Scores were recorded at 2 hours, 4 hours, and 6 hours postoperatively and the next morning [11]. Each measurement was divided by 10 to give a final score between 0 and 10.

Potential confounding factors that may have contributed to throat pain, such as type of airway ventilation device (e.g., endotracheal tube and laryngeal mask airway) and whether a throat pack was used, were also recorded.

### 2.6. Statistical Methods

Statistical analysis was performed using Stata version 14.0 (StataCorp 2015* Stata Statistical Software: Release 14*, College Station, TX: StataCorp LP.) and a* p *value < 0.05 was considered statistically significant. Continuous, parametric variables were presented as means with standard deviations, while categorical variables were presented as percentages. Comparison of VAS scores was performed using a Student's unpaired* t*-test. Comparison of potential airway confounders between groups was performed using Fisher's exact test.

## 3. Results

### 3.1. Participants' Characteristics

The mean ages of Moffett's solution and Cophenylcaine groups were 44.6 and 50.0 years of age, respectively, with 45% and 50% being male. There was no statistically significant difference in the two groups in terms of patient demographics and airway management for the operation. The majority of the patients in both groups received an endotracheal tube (80% and 90%, resp.), mostly without a throat pack (Table 1). In terms of operative characteristics, the two groups did not differ significantly in the types of procedure performed (septoplasty and turbinoplasty 60% versus 50%, p=0.71; septoplasty, turbinoplasty, and FESS 30% in both arms, p=0.69; and FESS alone 10% versus 20%, p=0.58).

### 3.2. Main Results

**Figure 1:**
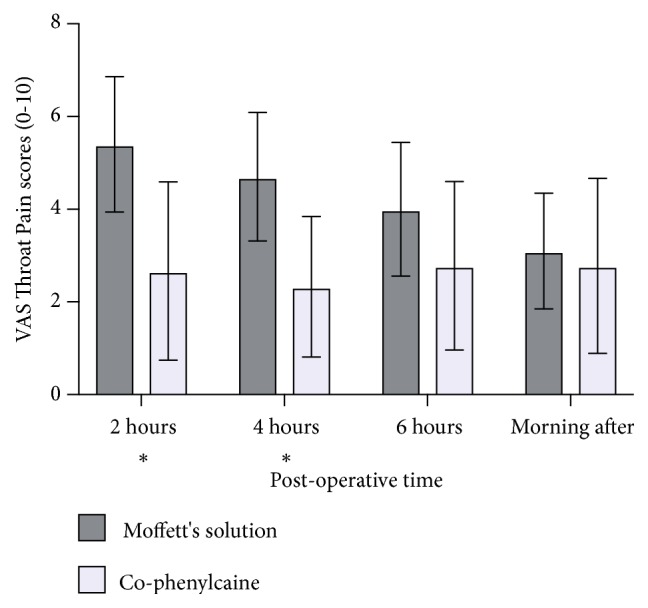
Postoperative visual analogue scores (0-10) for throat pain. *∗* denotes statistical significance (p<0.05). At 2 hours, p=0.03. At 4 hours, p=0.04. Error bars represent 95% confidence intervals.

There were statistically significant differences in the VAS scores at 2 hours postoperatively (mean of 5.40 ± 3.12 versus 2.67 ± 2.69, p=0.03) and at 4 hours (mean of 4.70 ± 2.96 versus 2.33 ± 2.12, p=0.04) (Figure 1). However, while the Moffett's solution group continued to report higher scores of throat pain, the differences were not significant in the two groups at 6 hours postoperatively and in the morning after surgery (Table 2).

No systemic side effects of either Moffett's solution or Cophenylcaine were observed in the duration of this study. Procedures included various combinations of functional endoscopic sinus surgery (FESS), septoplasty, and inferior turbinoplasty.

## 4. Discussion

Moffett's solution remains a popular choice for the preparation of the surgical field in sinonasal surgery due to its efficacy and safety in achieving profound decongestion [4]. It decreases intraoperative bleeding by vasoconstriction and allows improved operative access and visualisation by decongesting nasal mucosa [12]. The combination of cocaine and adrenaline synergistically acts on both *α*1 and *α*2 adrenoeceptors in nasal vasculature. By contrast, Cophenylcaine (phenylephrine and oxymetazoline) acts only on *α*1 adrenoreceptors. *α*1 adrenoreceptors chiefly innervate the arterial system and *α*2 adrenoreceptors chiefly innervate the venous system. Although the surgical conditions created using Moffett's solution and Cophenylcaine have not previously been directly compared in the literature, Moffett's solution has a theoretical advantage over Cophenylcaine [13]. In the present study, the solution was administered via the MADgic atomiser, which allows the solution to be distributed as a fine mist, effectively reaching most exposed surfaces of the nasal mucosa.

The present study found that the use of Moffett's solution in sinonasal surgery causes a statistically significant increase in throat pain when compared to its alternative, Cophenylcaine, in the postoperative period for up to four hours. Although throat pain has been reported in the literature as a minor side effect of Moffett's solution, to our knowledge, this is the first and only study to examine this specific relationship in detail [4].

Cocaine, or scientifically benzoylmethylecgonine, is an ester. When applied topically, it exerts its vasoconstrictive and analgesic effects via two different pathways. Its vasoconstrictive property is affected indirectly by presynaptic blockade of catecholamine reuptake, thereby increasing stimulation of the presynaptic adrenergic receptor [14, 15]. *α*1 adrenergic receptors when stimulated are responsible for arterial vasoconstriction, decreasing flow to capillary networks, while the stimulation of *α*2 adrenergic receptors decreases flow to venous sinusoids, thus causing decongestion [16]. When administered in conjunction with adrenaline, the two agents act synergistically to cause intense vasoconstriction. Furthermore, the combined administration of cocaine and adrenaline, as in Moffett's solution, has been shown by serial blood tests and liquid gas chromatography to reduce the systemic absorption of cocaine, hence improving its safety profile [17, 18]. Cocaine exerts its analgesic effects by blocking sodium channels along the axons of sensory nerves, dampening pain signal generation and propagation. The bicarbonate in Moffett's solution raises the pKa of the solution, which significantly increases the diffusion of cocaine across the nasal mucosa and axon membranes [15].

Throat pain is the second most common minor side effect of Moffett's solution after transient tachycardia [4]. Anecdotally, our group has noted that the throat pain experienced postoperatively can be severe and can have a significant impact on postoperative pain management. As this study has demonstrated, patients who have received topical Moffett's solution reported significantly higher scores in throat pain following surgery.

A number of anaesthetic confounding factors could potentially also lead to the complaint of throat pain postoperatively, including the choice of airway (ETT versus laryngeal mask), the use of throat packs, and trauma related to securing the airway. However, in this study, subgroup analysis demonstrated that the two arms had statistically similar rates of endotracheal intubation and throat pack use.

We postulate that the passage of excess Moffett's solution through the nasopharynx results in prolonged pooling in the oropharynx, causing profound and prolonged decongestion of the oropharyngeal mucosa, resulting in temporary superficial ischaemic changes, thereby leading to the complaint of postoperative throat pain. We hypothesise that pain in the nose following Moffett's solution administration is not seen as the solution and is only in transient contact with nasal mucosa before being either absorbed or passing through to the oropharynx. The effect may also be diminished by the rich vascular supply of the nose, compared to the oropharynx, which reduces the likelihood of ischaemic pain. However, we emphasize that this is the author's speculation and further research may be required to determine the true cause of postoperative throat pain in this context.

Such postapplication pooling in the oropharynx could potentially be prevented by various methods, such as the use of a reduced volume of solution, use of nebulised solution instead of sprayed solution, use of a postnasal balloon to prevent posterior passage, and immediate suctioning of excess solution from the oropharynx. Based on the results of this study, we propose that further studies to assess such methods are warranted.

### 4.1. Limitations

The current study has several limitations. Firstly, this pilot study is a small cohort study and the patients were not randomised. As a result, the investigators applying the solution and those recording the results were not blinded. As the two groups of patients underwent surgery under two different surgeons, there is a potential for the surgeons' approach to contribute to the observed results. As the patients received various combinations of septoplasty, turbinoplasty, and FESS, there is a potential for the differences in procedures to contribute to the observed results. The duration of the procedures was not analysed in the present study and may also play a role in postoperative throat pain. In this study, there was a high usage of ETT compared to laryngeal mask. Although results were similar in both forms of airway, the study was not sufficiently powered to compare these two groups. Lastly, postoperative throat pain was a self-reported outcome measure. This is subject to various factors affecting the patients' perception and tolerance of pain.

### 4.2. Conclusion

Moffett's solution is an efficacious, safe, and commonly used agent in preparing the surgical field for sinonasal surgery. This study is the first to show a statistically significant increase in throat pain up to 4 hours after the use of Moffett's solution in sinonasal surgery when compared to an alternative, Cophenylcaine. Based on these results, the authors propose that future studies designed to investigate methods of preventing postoperative throat pain associated with Moffett's solution are warranted.

## Figures and Tables

**Table 1:** Patient demographics and airway preparation.

	Moffett's solution (n=20)	Cophenylcaine (n=10)	*∗*p value (odds ratio, 95% CI)
Mean age	44.6 (SD 18.1)	50.0 (SD 19.0)	0.63

Male	9 (45%)	5 (50%)	1.00 (1.22, 0.27-5.60)

*Airway* Endotracheal tube (ETT), n (%)	16 (80%)	9 (90%)	0.64 (0.44, 0.04-4.61)

Laryngeal mask airway (LMA), n (%)	4 (20%)	1 (10%)	0.64 (0.44, 0.04-4.61)

Throat pack, n (%)	3 (15%)	2 (20%)	1.00 (0.71, 0.10-5.10)

The majority of the patients in both arms received endotracheal intubation and did not receive throat packs. The two arms did not differ significantly in airway preparation.

**Table 2:** Postoperative visual analogue scale for throat pain.

	Moffett's solution (n=20) Mean (SD, 95% CI)	Cophenylcaine (n=10) Mean (SD, 95% CI)	p value
*VAS score for throat pain* 2 hours postoperatively	5.40 (3.12, 4.03-6.77)	2.67 (2.69, 0.91-4.42)	0.03

4 hours postoperatively	4.70 (2.96, 3.40-6.00)	2.33 (2.12, 2.25-2.41)	0.04

6 hours postoperatively	4.00 (3.08, 2.65-5.35)	2.78 (2.54, 1.12-4.44)	0.31

Morning after operation	3.10 (2.67, 1.93-4.27)	2.78 (2.64, 1.06-4.50)	0.77

Statistically significant differences in postoperative VAS score for throat pain were observed at 2 and 4 hours (p=0.03 and p=0.04).

## Data Availability

Data is available upon request.

## Appendix

See Tables 1 and 2 and Figure 1.

### Summary

- Moffett's solution consisting of cocaine, adrenaline, and bicarbonate is a safe and effective nasal decongestant for endoscopic sinus surgery.
- Major side effects of Moffett's solution are very rare.
- Postoperative throat pain associated with the use of Moffett's solution has been reported anecdotally but has not been quantified.
- This study shows that the use of Moffett's solution when compared to Cophenylcaine in preparation of sinonasal surgery is associated with increased postoperative throat pain up to 4 hours postoperatively.
- Future studies may investigate means by which postoperative throat pain can be minimized.
